# Adaptive sequence convergence of the tumor suppressor ADAMTS9 between small-bodied mammals displaying exceptional longevity

**DOI:** 10.18632/aging.101180

**Published:** 2017-02-26

**Authors:** Matthew J. Lambert, Christine V. Portfors

**Affiliations:** ^1^ School of Biological Sciences, Washington State University, Vancouver, WA 98686, USA

**Keywords:** ADAMTS9, convergent evolution, longevity, naked mole-rat, microbats

## Abstract

Maximum lifespan varies by two orders of magnitude across mammals. How such divergent lifespans have evolved remains an open question, with ramifications that may potentially lead to therapies for age-related diseases in humans. Several species of microbats as well as the naked mole-rat live much longer than expected given their small sizes, show reduced susceptibility to neoplasia, and largely remain healthy and reproductively capable throughout the majority of their extended lifespans. The convergent evolution of extreme longevity in these two groups allows for the opportunity to identify potentially important aging related genes that have undergone adaptive sequence convergence in these long-lived, yet small-bodied species. Here, we have tested 4,628 genes for evidence of convergence between the microbats and naked mole-rat. We find a strong signal of adaptive sequence convergence in the gene A disintegrin-like and metalloprotease with thrombospondin type 1 motifs 9 (ADAMTS9). We also provide evidence that the shared substitutions were driven by selection. Intriguingly, ADAMTS9 is a known inhibitor of the mTor pathway and has been implicated in several aging related processes.

## INTRODUCTION

For several decades, it has been well recognized that there is strong correlation between lifespan and body mass [1,2], with larger species typically living longer than smaller species. There are, however, several species that violate this general rule, living much longer than expected given their small size and high metabolic rates. Of particular interest are the microbats, several species of which demonstrate longer maximum life-spans than any other mammals when controlling for body size [3]. In addition to their exceptional longevity, microbats appear to be resistant to neoplasia [4, 5] and remain healthy and reproductively capable throughout the majority of their lives [6].

Much like the microbats, *Heterocephalus glaber* (naked mole-rat) lives approximately three times longer than expected given its small size [7], is remarkably resistant to neoplasia [8-10] and displays no symptoms of aging well into its second decade [11].

Although once thought to be rare, there have been numerous recent studies demonstrating adaptive sequence convergence between a variety of species displaying convergent traits. These studies have highlighted genes that have been repeatedly targeted during the evolution of a given trait. For example, the evolution of echolocation in bats and toothed whales appears to be driven, in part, by common mutations in the genes *Prestin* [12, 13] and *Cdh23* [14, 15]; cardiac glycoside toxin resistance in numerous disparate vertebrates and invertebrates can be explained by identical amino acid substitutions in the enzyme Na+/K+-ATPase [16]; and the repeated convergent evolution of the sodium ion transporter NaV1.7 in hibernating mammals and mole-rats is believed to impart insensitivity to the accumulation of CO2 [17]. These studies, and many others, demonstrate that common selective pressures can drive common mutations in relevant genes.

The evolution of extreme longevity in microbats and the naked mole-rat is likely attributable to a lack of extrinsic sources of mortality in these species. Bats, being nocturnal and capable of flight, generally contend with few predators. Likewise, the naked mole-rat lives in subterranean burrows where the risk of predation is low. Several theories of aging suggest that a lack of extrinsic sources of mortality will result in selection for longer lifespan [8, 18]. For example, according to the antagonistic pleiotropy (AP) theory of aging, a mutation can be beneficial during development, but have late-onset deleterious effects [19]. AP is expected to be more prevalent in species with high levels of extrinsic mortality since most individuals are unlikely to survive long after reaching sexual maturity, therefore there will be little pressure to select against the deleterious effects that manifest later in life. Also, the disposable soma theory of aging suggests that there exists a trade-off between growth/development and repair/maintenance [20]. In species that contend with many predators, it should be beneficial to allocate resources to grow and develop as quickly as possible rather than to invest in repair and maintenance since longevity is already unlikely. According to both theories, for species that contend with numerous extrinsic sources of mortality, the decline in fitness due to aging is minimal, so selection is inefficient at promoting mutations that increase longevity. However, for species that exist in relatively safe niches, like microbats and the naked mole-rat, the strength of selection to delay senescence will be much stronger, as individuals that live longer will have higher lifetime reproductive fitness. We hypothesize that the pressure to delay senescence shared by microbats and naked mole-rat may have led to convergent sequence evolution in key longevity promoting genes. The identification of genes that have undergone convergent evolution in these long-lived species would provide a better understanding of the genetics of longevity and could potentially identify therapeutic targets for cancer and other age-related illnesses.

Here we tested for adaptive convergent sequence evolution between microbats and the naked mole-rat in almost 5,000 genes conserved across a wide-range of mammals. We found that A disintegrin-like and metalloprotease with thrombospondin type 1 motifs 9 (ADAMTS9) displays numerous convergent substitutions between the long-lived species that were likely driven by positive selection.

## RESULTS

**Figure 1:**
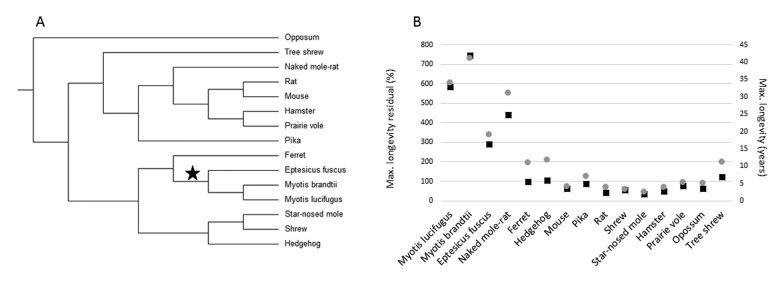
Small-bodied mammals that display exceptional longevity (**A**) The accepted tree topology for the 15 species included in this study. (**B**) Maximum longevity residual (left axis, black boxes) and maximum longevity (right axis, grey circles) of the 15 species. Maximum longevity residual (tmax) is the percentage of the expected maximum longevity given adult body size (*M*), derived from the mammalian allometric equation: *tmax* = 4.88*M*^0.153^ [30].

The goal of this study was to determine if any genes display evidence of adaptive sequence convergence between long-lived microbats and the naked mole-rat. We tested for convergence in 4,628 1:1 ortholog groups between 3 microbat species (*Myotis lucifugus, Myotis brandtii* and *Eptesicus fuscus*), and the naked mole-rat. Additionally, as outgroup species, we included sequences from 11 small-bodied rodents and laurasiatherian mammals with at least acceptable longevity data curated in the AnAge database [21]. The relationship between the 15 species is shown in the phylogeny in Figure 1a. Importantly, none of these additional species demonstrate exceptional longevity (Fig. 1b). The requirement that sister species to microbats and naked mole-rat be small-bodied and not long-lived allowed us to control for body size and phylogeny while giving us the opportunity to test for convergence in the long-lived species as compared to normal aging species.

**Figure 2:**
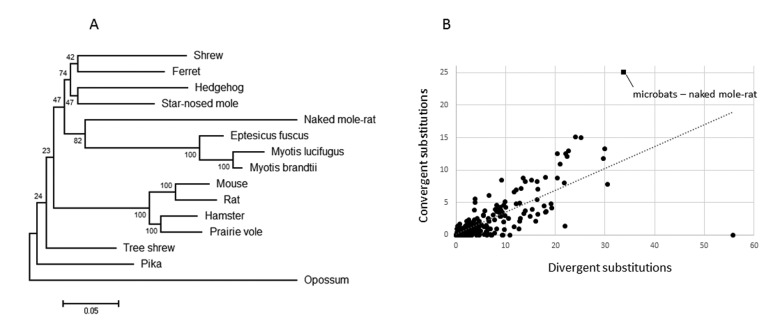
Evidence for ADAMTS9 sequence convergence in long-lived mammals (**A**) Maximum-likelihood gene tree constructed using the best fitting nucleotide substitution model (GTR+G) demonstrates a monophyletic grouping of the long-lived mammals. (B) Plot of the numbers of divergent versus convergent substitutions occurring between all independent pairs of branches of the species tree. The microbat-naked mole-rat comparison has the highest level of convergence as well as the largest distance from the trendline.

Initially, we constructed maximum likelihood gene trees based on nucleotide alignments for each of the 4,628 ortholog groups. These trees were then parsed programmatically, searching for instances in which the long-lived mammals (naked mole-rat, *Myotis davidii*, *Myotis lucifugus* and *Eptesicus fuscus*) formed a monophyletic group, to the exclusion of their true closest relatives (rodents and carnivores/insectivores for the naked mole-rat and microbats, respectively). We found that only one gene, ADAMTS9, violated the known species tree in such a manner (Fig. 2a), suggesting sequence convergence between the naked mole-rat and microbats occurred during the evolution of this gene. To determine which sites were responsible for the observed grouping of the long-lived species, we used a maximum likelihood approach to reconstruct all ancestral amino acid sequences at interior nodes of the accepted phylogeny. These ancestral sequences were used to map and count all pairwise convergent substitutions (mutations from the ancestral state that lead to identical amino acids) shared between the microbats and naked mole-rat. Although, the microbats and naked mole-rat share 21 convergent substitutions in this gene (Table 1), which is far more than any other instance of sequence convergence described to date, it may be possible that this represents a normal level of background convergence if for example ADAMTS9 is rapidly evolving. However, Castoe et al. [22] developed a method to generate an empirical null distribution for the expected level of convergence for a given gene, provided a sufficient number of taxa are included. Briefly, the number of divergent (mutations that lead to different amino acids at homologous sites in the species being compared) and convergent substitutions are estimated for every pair of branches in a phylogeny. It has previously been demonstrated that the number of divergent substitutions reliably predict the number of convergent substitutions, therefore excess convergence between any two branches would appear as an outlier with a higher convergent to divergent substitution ratio than all other pairwise comparisons between branches [22]. This method allowed us to distinguish between rapid evolution and potential adaptive convergence. To test this, we used the program Grand-conv [23, 24], which estimates the posterior numbers of convergent and divergent substitutions shared between all pairs of branches in the given phylogeny. We found that the convergence between the naked mole-rat and microbats exceeded expectations given the phylogeny (Fig. 2b), again suggesting sequence convergence between the long-lived species. In addition, using the methods of Zhang and Kumar [25], we found that the level of convergence between microbats and naked mole-rat exceeds random expectations (p < .001), indicating a significant amount of convergence.

**Table 1:** ADAMTS9 convergent sites The numbers at the top of each column indicate the amino acid position in the alignment.

	54	236	244	324	362	512	558	565	775	838	884	890	1110	1314	1319	1489	1497	1597	1675	1765	1933
Naked mole-rat	V	R	R	V	P	S	D	Q	A	K	V	R	Q	R	R	R	E	R	A	S	T
Eptesicus fuscus	V	R	R	V	P	S	D	Q	A	K	V	R	Q	R	R	R	E	R	A	S	T
Myotis brandtii	V	R	R	V	P	S	D	Q	A	K	V	R	Q	R	R	-	-	R	A	S	T
Myotis lucifugus	V	V	Q	V	P	S	D	Q	A	K	V	R	Q	R	R	R	E	R	A	S	T
Rat	I	K	K	I	S	P	-	-	T	S	I	R	E	Q	T	K	D	Q	T	N	L
Mouse	I	K	K	I	Y	P	H	R	T	S	I	K	E	Q	T	K	D	Q	T	N	K
Prairie vole	I	K	K	I	S	P	H	R	T	S	I	K	E	Q	T	K	D	Q	T	N	K
Hamster	I	K	K	I	S	P	H	R	T	N	I	K	E	Q	R	K	D	Q	T	N	K
Star-nosed mole	I	K	K	I	S	S	P	R	T	N	V	K	E	Q	S	K	D	K	T	K	K
Shrew	-	K	K	I	S	X	L	R	T	N	V	K	E	Q	S	X	X	K	T	L	K
Ferret	I	K	K	I	S	P	-	R	T	N	I	K	E	Q	S	K	D	K	T	E	K
Tree shrew	V	K	K	I	X	P	X	X	T	X	-	K	E	Q	S	K	E	K	X	X	K
Hedgehog	-	S	K	I	S	Y	H	R	X	N	V	K	E	Q	D	K	D	K	T	N	K
Opossum	-	K	R	V	A	P	H	R	T	N	I	S	A	R	R	K	D	K	T	K	K
Pika	I	S	K	V	S	P	H	R	T	N	I	K	E	Q	M	K	D	K	A	Q	K

Notably, the addition of the moderately long-lived rodent, Cavia porcellus (guinea pig), and megabats, Pteropus alecto and Pteropus vampyrus, disrupted the monophyletic grouping of the naked mole-rat and micro-bats in maximum-likelihood gene trees based on amino acid alignments (Not shown). However, even with the inclusion of these moderately long-lived mammals we still found that the naked mole-rat and microbats share 16 convergent substitutions (Supplemental Table 1), which remains significant using the method of Zhang and Kumar [25]. Furthermore, estimates of posterior numbers of divergent versus convergent changes still suggest sequence convergence between the naked mole-rat and microbats even when the megabats and guinea pig were included (Supplemental Fig. 1).

**Figure 3:**
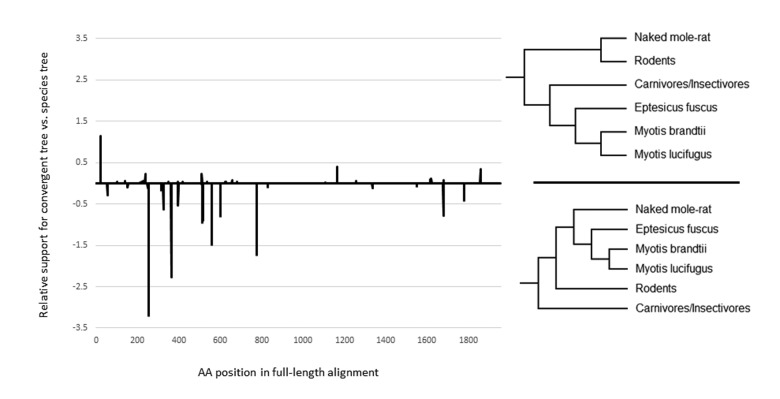
The relationship between sequence convergence and positive selection Relative support for convergent tree and species tree topologies for all positively selected sites along the *ADAMTS9* gene sequence. The values are the difference between site-wise log likelihood scores for the species tree and the convergent tree. Negative values indicate more support for the convergent tree.

Lastly, to test if the observed sequence convergence may be due to positive selection, we employed a unique multistep approach. First, all sites with significant evidence for positive selection along the ancestral microbat branch (indicated by a star in Fig. 1a) were identified with the program TreeSAAP [26], producing a list of 77 sites. These 77 sites were then extracted from the original alignment and used to create a new alignment that consisted only of the sites with evidence of positive selection in microbats. Next, we calculated site-wise log-likelihood values for the convergent gene tree (Fig. 2a) and compared these to site-wise log likelihood values for the accepted species tree (Fig. 1a), using the positively selected sites alignment. If the observed convergence was due to chance, we would expect the positively selected sites to support the accepted species topology. However, we found that the opposite is true, the sites with evidence of positive selection strongly favored the convergent topology (Fig. 3). Indeed, an approximately unbiased test suggests that the convergent tree was much more likely than the accepted species tree (P = .886 and P = .114, respectively), suggesting that the convergence between the long-lived species was driven by selection.

## DISCUSSION

We tested 4,628 genes for evidence of adaptive sequence convergence between long-lived, small-bodied mammals. We found evidence for an enrichment of convergent substitutions between the microbats and the naked mole-rat in the gene ADAMTS9. ADAMTS9 is the most widely conserved member of the ADAMTS family and has recently been reported to be a novel tumor suppressor that is downregulated in several varieties of human cancer [27]. Intriguingly, ADAMTS9 inhibits tumor growth by blocking the mTOR pathway [28], which has long been known to be associated with aging [29]. In addition to its role in tumor suppression, ADAMTS9 has also been implicated in several age-related conditions including arthritis [30], type 2 diabetes [31, 32], age-related macular degeneration [33, 34] and menopause [35]. Furthermore, in C. elegans the loss of GON-1, the roundworm homolog of ADAMTS9, alters lifespan and promotes dauer formation [36]. These effects are likely due to modified insulin and insulin-like ortholog secretion and altered insulin/IGF-1 signaling, which is also known to contribute to aging [37].

Although, it may be possible that the observed convergent changes shared by microbats and the naked mole-rat may be the product of some non-adaptive force rather than selection for increased longevity, several lines of evidence suggest otherwise. First, the convergent substitutions are distributed along the length of the coding sequence, eliminating gene conversion or alternate exon usage as possible causes. Second, the convergent topology was strongly favored when only sites with evidence of positive selection occurring on the long-lived microbat branch were considered, suggesting that the convergence was indeed driven by selection. Finally, ADAMTS9 has previously been implicated in several aging processes and age-related diseases [27, 30-37], supporting the hypothesis that modulation of ADAMTS9 function alters lifespan. Together, this evidence suggests that ADAMTS9 has been repeatedly targeted by selection for increased longevity in microbats and the naked mole-rat.

As yet, we can provide no explanation for how the convergent evolution of ADAMTS9 has promoted longevity in microbats and the naked mole-rat. However, it is rather intriguing that ADAMTS9 is a known inhibitor of the mTor pathway. The hyperfunction theory of aging posits that aging is due to the prolonged activity of growth-promoting pathways, such as mTor [38]. According to this theory, aging itself is not a program, but rather aging is a harmful continuation of developmental programs after growth has ended [39]. This hypothesis is supported by several lines of evidence that have identified mTor activity as a driver of senescence [29, 40-43]. Therefore, it is a distinct possibility that the convergent evolution of ADAMTS9 in long-lived species may have altered this gene's function such that it now serves to slow the effects of mTor, preventing the declines normally associated with aging. Indeed, the author of the hyperfunction theory has recently speculated that the extraordinary longevity of microbats and the naked mole-rat may be due to selection for a “decelerator of mTor” [44].

Given the role of ADAMTS9 in such a wide range of age-related conditions, its direct effect on lifespan in C. elegans and its ability to modulate mTOR and insulin/IGF-1 signaling, it is likely that the convergent evolution of ADAMTS9 may be, in part, responsible for the exceptional longevity and resistance to neoplasia found in microbats and the naked mole-rat.

## MATERIALS AND METHODS

Ensembl Compara [45] was used to identify all 1:1 orthologs from the species Myotis lucifugus, Pteropus vampyrus, Erinaceus Europaeus, Mustela putorius, Sorex araneus, Mus musculus, Rattus norvegicus, Ochotona princeps, Cavia porcellus and Monodelphis domesticus. This generated a list of 5109 1:1 ortholog groups from these species. Amino acid and coding sequences for the species listed above were downloaded from Ensembl version 84. For the species Myotis brandtii, Eptesicus fuscus, Miniopterus natalensis, Pteropus alecto, Condylura cristata, Mesocricetus auratus, Microtus ochrogaster and Heterocephalus glaber, orthologs were identified with a recriprocal best hit approach. For example, the naked mole-rat orthologs were identified by first using blastp to align the 5,109 Cavia porcellus protein sequences from Ensembl against all naked mole-rat refSeq sequences. The top naked mole-rat hits were then aligned against all Cavia porcellus protein sequences from Ensembl. All instances in which the reciprocal blast alignments identified the same sequences were included for further study, all others were discarded. This reduced the number of 1:1 ortholog groups to 4,978.

Amino acid sequences for each of the 4,978 1:1 ortholog groups were aligned with Muscle [46]. TrimAl [47] was used to filter alignments with mean percentage identity below 60%. This left 4,628 high quality multiple sequence alignments. Pal2Nal [48] was then used to create codon oriented nucleotide alignments for the 4,628 ortholog groups. We then constructed Maximum-likelihood gene trees in PhyML 3.0 [49] using the best fitting nucleotide substitution model for each gene as indicated by jModelTest 2 [50]. A custom perl script was used to analyze the resulting Newick files, searching for monophyletic groupings of the long-lived species.

PAML, specifically the program CodeML, [51] was used to reconstruct ancestral amino acid sequences at all interior nodes of the species tree for ADAMTS9. We then used custom Perl scripts to map and count all convergent substitutions occurring between the naked mole-rat and microbat branches. Since detection of convergent and divergent substitutions is critically dependent upon alignment quality we used PRANK [52], a phylogeny aware alignment tool, to generate a second ADAMTS9 multiple sequence alignment. Differences between the Muscle and PRANK alignments were minimal and both recovered the same 21 convergent substitutions between microbats and naked mole-rat.

Both the Muscle and PRANK ADAMTS9 amino acid multiple sequence alignments, the accepted tree topology and the program Grand-conv were used to estimate the posterior numbers of convergent and divergent substitutions. The two alignments produced identical results. To determine which sites were subject to positive selection along the microbat lineage we used the program TreeSAAP. The ADAMTS9 nucleotide multiple sequence alignment and the accepted species tree were used as input. We only considered radical substitutions (categories 6-8) with Z-scores greater than 3.09. PhyML was used to calculate site-wise log likelihood values for both the convergent tree and a constrained species tree using a multiple sequence alignment containing only sites with evidence of positive selection as indicated by TreeSAAP. An approximately unbiased test comparing the likeliness of the convergent and species trees was conducted in Consel [53].

## SUPPLEMENTARY MATERIALS TABLES AND FIGURES


