# Supplementary material for: Adaptive sequence convergence of the tumor suppressor ADAMTS9 between small-bodied mammals displaying exceptional longevity
Source: Aging (Albany NY). 2017 Feb 26;9(2):573–81. doi: 10.18632/aging.101180 (PMC5361682; doi:10.18632/aging.101180)
Supplement: Supplementary file 1 [file aging-09-573-s001.pdf]

SUPPLEMENTARY MATERIAL

Supplemental Table 1. ADAMTS9 convergent sites. The numbers at the top of each column indicate the amino acid position in the alignment.

	54	236	244	362	512	775	838	890	1110	1342	1489	1497	1597	1675	1765	1933
Naked mole-rat	V	R	R	P	S	A	K	R	Q	G	R	E	R	A	S	T
Eptesicus fuscus	V	R	R	P	S	A	K	R	Q	G	R	E	R	A	S	T
Myotis brandtii	V	R	R	P	S	A	K	R	Q	G	-	-	R	A	S	T
Myotis lucifugus	V	V	Q	P	S	A	K	R	Q	G	R	E	R	A	S	T
Pteropus vampyrus	I	K	K	S	P	T	N	K	E	S	K	D	K	T	N	K
Pteropus alecto	I	K	K	S	P	T	N	K	E	S	K	D	K	T	N	K
Guinea pig	Q	D	F	L	P	T	N	K	E	S	K	D	Q	T	N	K
Mouse	I	K	K	Y	P	T	S	K	E	S	K	D	Q	T	N	K
Rat	I	K	K	S	P	T	S	R	E	S	K	D	Q	T	N	L
Hamster	I	K	K	S	P	T	N	K	E	S	K	D	Q	T	N	K
Prairie vole	I	K	K	S	P	T	S	K	E	S	K	D	Q	T	N	K
Pika	I	S	K	S	P	T	N	K	E	G	K	D	K	A	Q	K
Shrew	-	K	K	S	X	T	N	K	E	N	X	X	K	T	L	K
Ferret	I	K	K	S	P	T	N	K	E	G	K	D	K	T	E	K
Tree shrew	V	K	K	X	P	T	X	K	E	S	K	E	K	X	X	K
Hedgehog	-	S	K	S	Y	X	N	K	E	S	K	D	K	T	N	K
Star-nosed mole	I	K	K	S	S	T	N	K	E	N	K	D	K	T	K	K
Opossum	-	K	R	A	P	T	N	S	A	N	K	D	K	T	K	K

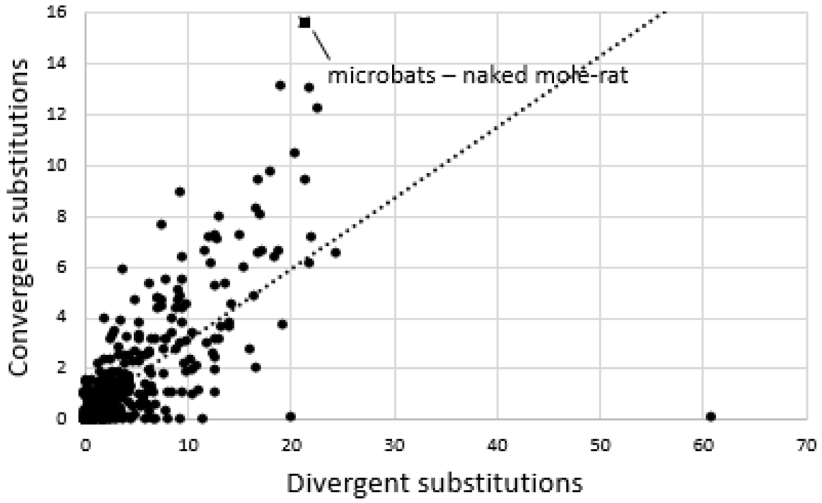

Supplemental Figure 1. Evidence for ADAMTS9 sequence convergence in long-lived mammals. Plot of the numbers of divergent versus convergent substitutions occurring between all independent pairs of branches of the species tree.
